# Organ-on-a-chip technology for nanoparticle research

**DOI:** 10.1186/s40580-021-00270-x

**Published:** 2021-07-08

**Authors:** Shawn Kang, Sunghee Estelle Park, Dan Dongeun Huh

**Affiliations:** 1 Department of Bioengineering, University of Pennsylvania, 210 S 33rd St., Philadelphia, PA 19104 USA; 2 NSF Science and Technology Center for Engineering Mechanobiology, University of Pennsylvania, Philadelphia, PA 19104 USA; 3 Institute for Regenerative Medicine, Perelman School of Medicine, University of Pennsylvania, Philadelphia, PA 19104 USA

**Keywords:** Organ-on-chip, Microfluidics, Microphysiological systems, Nanoparticles, Nanomedicine, Nanotherapeutics, Drug delivery

## Abstract

The last two decades have witnessed explosive growth in the field of nanoengineering and nanomedicine. In particular, engineered nanoparticles have garnered great attention due to their potential to enable new capabilities such as controlled and targeted drug delivery for treatment of various diseases. With rapid progress in nanoparticle research, increasing efforts are being made to develop new technologies for in vitro modeling and analysis of the efficacy and safety of nanotherapeutics in human physiological systems. Organ-on-a-chip technology represents the most recent advance in this effort that provides a promising approach to address the limitations of conventional preclinical models. In this paper, we present a concise review of recent studies demonstrating how this emerging technology can be applied to in vitro studies of nanoparticles. The specific focus of this review is to examine the use of organ-on-a-chip models for toxicity and efficacy assessment of nanoparticles used in therapeutic applications. We also discuss challenges and future opportunities for implementing organ-on-a-chip technology for nanoparticle research.

## Introduction

Remarkable progress in nanotechnology over the last decades has greatly advanced the field of nanomedicine. At the center of this active area of research is the development of engineered nanoparticles for a wide array of biomedical applications ranging from medical imaging [1, 2] to biosensing and point-of-care diagnostics [3, 4]. In particular, building upon major advances in chemistry and materials science, researchers have demonstrated various types of functionalized nanoparticles that enable highly tissue-specific delivery and controlled release of therapeutic payloads for treatment of cancer [5–7], respiratory diseases [8–10], neurological diseases [11, 12], acquired immunodeficiency syndrome (AIDS) [13, 14], cardiovascular diseases [15, 16], and ocular diseases [17–19]. With the rapid advancement of nanotechnology, engineered nanoparticles provide a basis for the development of novel therapeutics that represent the new frontiers of nanomedicine [20–23].

While considerable progress has been made in the design and production of various types of nanotherapeutics, clinical translational of these engineered nanomaterials continues to be a challenge. Among the major problems underlying this challenge is the limited predictive capacity of conventional preclinical methods for evaluating the safety and therapeutic efficacy of nanoparticles. With increasing evidence pointing to significant species-specific differences [24, 25], traditional approaches based on animal studies have come under increased scrutiny in recent years, questioning their ability to adequately represent human physiology and make accurate predictions of human responses to nanoparticles [26, 27]. On the other hand, the limitations of animal models have motivated efforts to improve the capabilities of traditional cell cultures to model the complexity of native systems by using three-dimensional (3D) culture scaffolds and new in vitro techniques for co-culture of multiple cell types [28].

Over the last decade, advances in this line of investigation have led to the development of a unique approach that exploits precision and controllability afforded by microengineering technologies to construct new types of complex in vitro models known as organs-on-a-chip [29, 30]. By providing a platform to emulate structural and functional complexity of human tissues and organ units in ways not possible in traditional cell culture, organs-on-chip offer a promising complementary approach to animal experimentation. In this topical review, we survey recent research efforts directed towards harnessing the power of these advanced model systems for the study of engineered nanoparticles. For focused discussion, this paper examines how organ-on-a-chip models can be implemented in preclinical assessment of nanoparticles designed specifically for applications in drug delivery.

## What are organs-on-a-chip?

Organs-on-a-chip, otherwise referred to as organ-chips, microphysiological systems (MPS), or tissue-chips, are microfabricated cell culture devices that are engineered to mimic the characteristics of multicellular tissue units or functional elements of organs [29–33]. As microengineered in vitro analogs of in vivo physiological systems, organs-on-a-chip are designed using a reductionist approach to identify and replicate the salient features of the specified organ in its native context [34]. For any given target organ, the process of creating its on-chip representation starts by reducing the organ to its most basic anatomical elements responsible for organ-specific physiological function (e.g., alveoli in the lung, nephrons in the kidney, osteons in the bone). The functional units of the organ are then closely examined to identify their key cellular constituents with distinct phenotype, structural organization of different cell and tissue types, and biochemical and/or mechanical cues that are present in their local microenvironment. These features are individually assessed for inclusion into the organ-chip model, which is followed by the design and fabrication of a microdevice needed to reproduce the identified features.

**Fig. 1:**
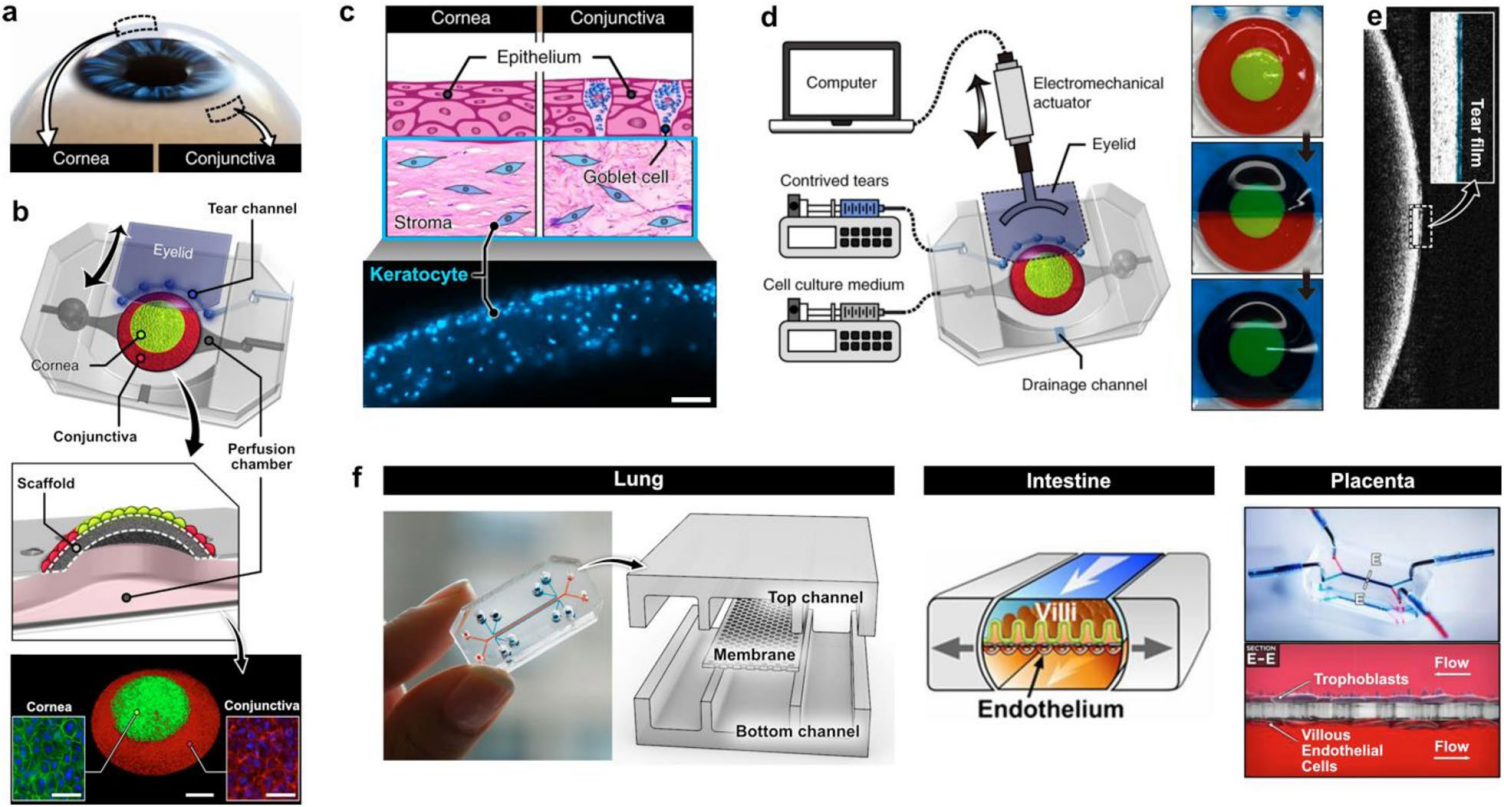
Design process of organ-on-chip models. **a** The blinking eye-on-a-chip mimics the human eye through a reductionist approach by targeting the ocular surface and its relevant components, the cornea and conjunctiva, as key functional units **b** and coculturing these cell types over a dome-shaped scaffold. Scale bars, 1 mm (middle), 50 µm (insets). **c** The underlying stroma is also modelled by embedding keratocytes into the scaffold. Scale bar, 100 µm. **d** The unique blinking property of the eye is recapitulated through electromechanical actuation, **e** which creates a tear film with physiological thickness. **f** This design principle has been used to model other organs such as the lung, intestine, and placenta in a conventional device consisting of overlapping perfusable microchannels separated by a semiporous membrane

For example, this reductionist design approach has been applied to constructing a human blinking eye-on-a-chip to model the ocular surface as one of the structural and functional units of the human eye (Fig. 1) [35]. To mimic the spatial arrangement of the cornea and the conjunctiva on the ocular surface (Fig. 1a), this system utilizes a dome-shaped convex polymeric scaffold for spatially patterned co-culture of primary human corneal and conjunctival epithelial cells (Fig. 1b). Three-dimensional microarchitecture of the stroma underlying the epithelial surface is also recreated by embedding primary human keratocytes in collagen hydrogel injected into the scaffold (Fig. 1c). Another unique feature of this system is its ability to mimic the dynamic mechanical environment of the ocular surface due to spontaneous eye blinking. To achieve this capability, a biomimetic eyelid made out of hydrogel is electromechanically actuated to slide back and forth over the surface of the microengineered ocular surface at defined speed and frequency (Fig. 1d). This mechanism also makes it possible to spread tears on the epithelial surface and form a thin tear film with physiological thickness (Fig. 1e).

Using the same design principle, researchers have demonstrated microengineered biomimetic models of various organs, including the lung [36, 37], intestine [38, 39], liver [40, 41], kidney [42, 43], brain [44, 45], and placenta [46, 47], among others. Many of these models have been established using a common device design that consists of two overlapping perfusable microchannels separated by a semipermeable polymeric membrane, which allows for co-culture of two or more cell types in fluidically independent chambers to mimic physiological tissue compartmentalization in vivo (Fig. 1f). When it comes to production of microdevices used in these models, soft lithography using elastomeric materials such as poly(dimethylsiloxane) (PDMS) [48, 49] has been the most popular method of choice. This approach has greatly facilitated the development of organ-chip systems in academic laboratory settings by enabling rapid prototyping of microfabricated devices with various design features that also offer optical transparency and gas permeability advantageous for imaging and cell culture, respectively [50].

From the standpoint of modeling functional complexity of in vivo systems, organs-on-a-chip have proven capable of reconstituting higher-level physiological functions and responses that result from complex biological interactions between multiple cell and tissue types [36, 51]. Recent studies have also demonstrated the development of various organ-chip-based specialized models that can reproduce complex pathophysiological processes for applications in mechanistic disease studies [52, 53] and drug discovery [31, 54, 55]. Moreover, great strides have been made over the last two decades in combining multiple organ-chip models to form integrated body-on-a-chip systems that can simulate physiological interactions between different organs and organ systems [51, 56, 57]. Most recently, increasing attention is being paid to the possibility of synergistically combining induced pluripotent stem cells (iPSCs) [58–60] and organoids [34, 61, 62] with organ-on-a-chip technology towards the development of patient-specific and more predictive and realistic preclinical models for biomedical, pharmaceutical, and environmental applications. Comprehensive review of recent advances in organ-on-a-chip technology is provided elsewhere [63, 64].

## Organ-on-a-chip models for preclinical assessment of nanoparticles

Evaluating the performance of engineered nanoparticles in an integrated, physiological context represents an area of increasing research attention to which organ-on-a-chip technology is well-poised to make unique contributions. As the main body of our review, this section examines novel capabilities and potential of organ-on-a-chip technology for preclinical assessment of nanoparticles. Our discussion focuses on representative studies from the recent literature that demonstrate the development of organ-chips specifically for the purposes of investigating (i) targeted delivery, (ii) therapeutic efficacy, and (iii) adverse health effects of engineered nanoparticles.

### In vitro modeling of targeted nanoparticle delivery in organ-chips

#### Investigating the effect of physicochemical properties of nanoparticles

Targeted delivery of nanotherapeutics is of utmost importance in nanomedicine that has been both a driver of research advances and a major challenge in the field. Nanoparticles carrying therapeutic payloads are designed to accumulate preferentially at disease sites in order to minimize unwanted off-target effects [65, 66]. On the basis of findings that this target-specific delivery can be achieved by engineering the properties of nanoparticles [67, 68], significant efforts have been made to develop organ-chip systems for in vitro modeling and analysis of how physicochemical characteristics of nanoparticles affect their transport, accumulation, and cellular uptake.

**Fig. 2:**
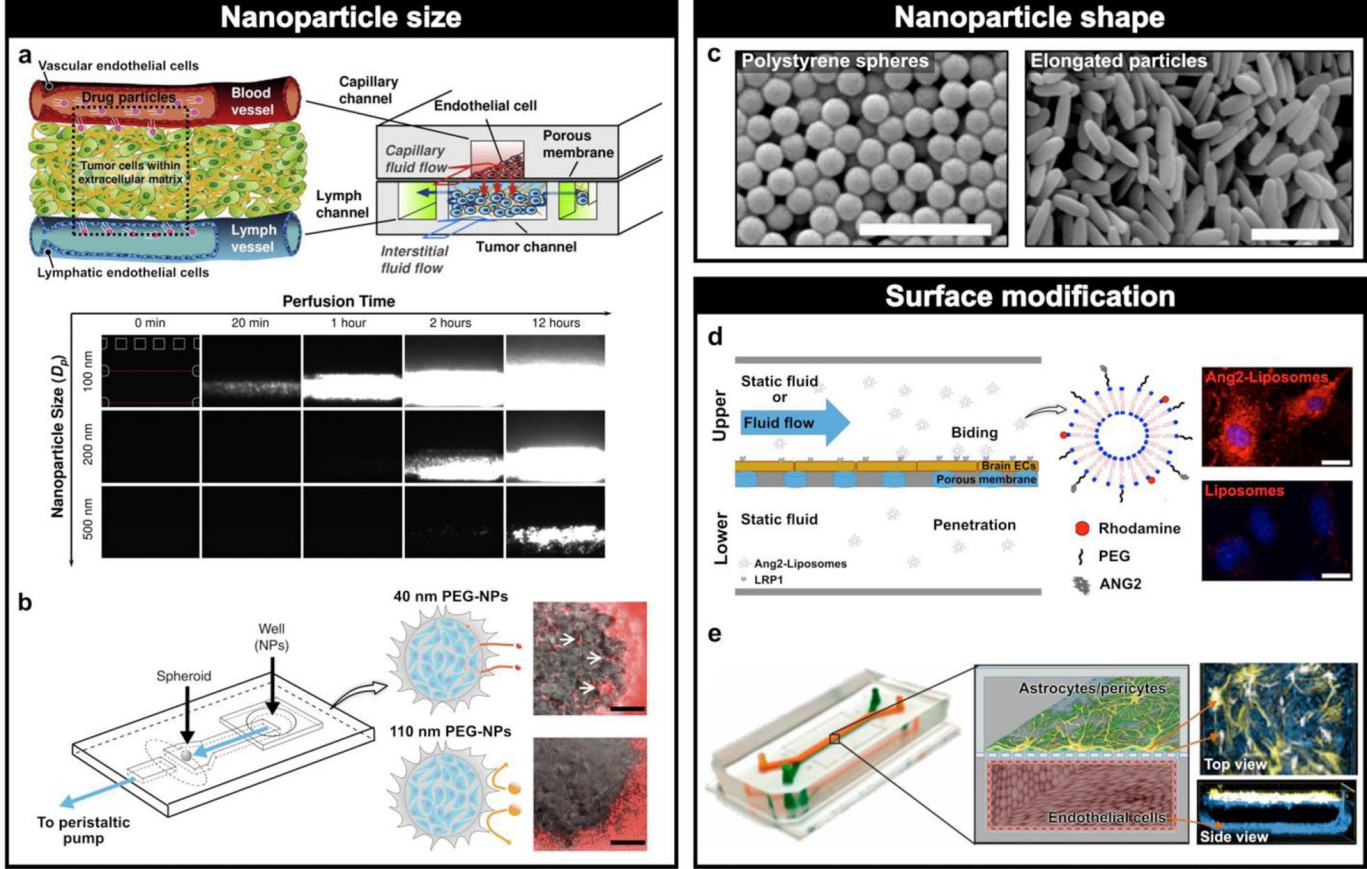
Effect of nanoparticle physicochemical properties. **a** A microengineered breast cancer tumor model mimicking the blood vessels, tumor tissue, and lymph vessels through three different types of channels demonstrated the correlation between smaller nanoparticle sizes and increased translocation across a porous membrane. **b** A tumor-on-a-chip model to assess nanoparticle accumulation at a tumor spheroid. Small particles were able to accumulate at the spheroids, whereas larger ones could not. Scale bars, 100 µm. **c** When anti-intracellular adhesion molecule-1 (ICAM) and immunoglobulin G antibody (IgG) coated nanorods and spheres were flown across a microfluidic device, nanorods demonstrated enhanced targeting and greater attachment to an endothelial monolayer compared to spheres. Scale bars, 1 µm. **d** A blood brain barrier (BBB) model with two overlapping channels separated by a porous membrane showed greater binding of liposomes modified with Angiopep-2 (Ang-2) to endothelial cells than non-functionalized ones. Scale bars, 10 µm. **e** A similar human BBB on-chip with human brain astrocytes and pericytes in the upper, central nervous system (CNS) compartment and brain-like microvascular endothelial cells in the lower, brain microvasculature compartment indicated an enhanced ability of Ang-2-coated quantum dots (QDs) to move across the CNS channel without affecting the barrier integrity compared to non-modified QDs

For example, Kwak et al. created a tumor-on-a-chip to model the trafficking of different sized nanoparticles in the complex tumor microenvironment [69]. To replicate a tumor tissue situated between lymphatic and blood vessels, this model was constructed in a multilayered microdevice assembled by bonding two sets of PDMS chambers to a semipermeable membrane made out of polycarbonates that contained 400 nm pores (Fig. 2a). In this 3D configuration, human microvascular endothelial cells were grown on the upper side of the membrane to model the tumor microvasculature, while MCF-7 human breast cancer cells were cultured in type I collagen hydrogel formed in the central chamber of the lower layer to mimic a tumor mass (Fig. 2a). Two additional channels were created adjacent to the tumor chamber in the lower layer to emulate lymphatic vessels. Importantly, this system provided a platform to measure translation and diffusive transport of fluorescently labeled nanoparticles, primarily used for imaging purposes, of varying diameters (100, 200, and 500 nm) from the vascular compartment to the microengineered tumor tissue into the lymphatics. For a particle size of 100 nm, relatively rapid trans-membrane transport and interstitial diffusion was observed, whereas larger particles with a diameter of 200 nm showed noticeably hindered transport (Fig. 2a). 500 nm particles, which were larger than the pore size, resulted in no penetration into the tumor channel. Although somewhat obvious, these results illustrated that nanoparticle delivery into complex 3D tissues and their drainage into lymphatic vessels are highly dependent upon the size of nanoparticles.

A similar approach has been used by Albanese et al. to investigate the effect of particle size on the accumulation of nanoparticles in tumor spheroids [70]. A tumor-on-a-chip model developed in this study featured a 3D microchannel design that permitted mechanical trapping of melanoma tumor spheroids in a multilayered PDMS device and their continuous perfusion with nanoparticle-containing media (Fig. 2b). When fluorescently labeled gold nanoparticles of varying diameters (40, 70, 110, and 150 nm) were introduced into the culture chamber, the model showed deep penetration of the spheroids by smaller particles (40 and 70 nm) and their accumulation in the interstitial spaces (Fig. 2b). By contrast, larger particles were excluded and not observed within the tumor constructs (Fig. 2b). Importantly, these findings illustrating the greater extent of tissue accumulation with smaller particle sizes were corroborated by a mice xenograft MDA-MB-435 model.

In addition to size, another important property that can be engineered to control nanoparticle delivery is shape. Although conventional nanoparticles are typically designed as spherical structures, recent research has demonstrated the development of nanoparticles with other shapes, such as nanorods and nano-discs [68, 71]. Organ-chips are emerging as useful in vitro platforms for studying how these new shapes affect nanoparticle transport and delivery, which represents an active area of investigation in nanoparticle research [72]. A good example of such systems is provided by a microfluidic model of the vascular endothelium established by using rat brain endothelial (RBE4) cells to examine cellular adhesion and internalization of different nanoparticle shapes [73]. By flowing spherical and rod-shaped nanoparticles (Fig. 2c) coated with anti-intracellular adhesion molecule-1 (ICAM-mAb) antibody or immunoglobulin G antibody (IgG) over the surface of the RBE4 monolayers, this study showed enhanced targeting and stronger attachment of nanorods to the endothelium compared to the spherical particles, highlighting the potential of unconventional nanoparticle shapes for more efficient and target-specific drug delivery.

Lastly, based on a growing body of evidence showing significant effects of surface chemistry on the behavior of nanoparticles [74, 75], microengineers have also begun to explore the use of organ-chips for studying how surface functionalization can be leveraged as a means to enhance nanoparticle delivery. Much of the work in this space has focused on looking into the penetration of nanoparticle drugs across the blood brain barrier (BBB). In one study, Papademetriou et al. created a BBB model by culturing brain microvascular endothelial cells (bEnd.3) in a microdevice consisting of two overlapping microchannels separated by a porous membrane (Fig. 2d) [76]. This system was used to model transport of liposomal drug nanocarriers engineered with a peptide ligand Angiopep-2 (Ang-2). Interestingly, when liposomes were introduced into the device maintained in static culture conditions, Ang-2-functionalized nanocarriers showed a significantly higher degree of binding to the apical surface of the brain endothelial cells compared to non-functionalized control (Fig. 2d). Under flow conditions, Ang-2 conjugation enhanced the ability of the nanoparticles to penetrate the endothelial monolayer without affecting barrier function. These results were consistent with the previously demonstrated ability of Ang-2 to facilitate delivery of anticancer drugs across the BBB [77, 78].

Another study conducted by Park et al. developed a human BBB-on-a-chip using the same device architecture [79]. In this system, primary human brain astrocytes and pericytes were cocultured on the upper side of the membrane to represent tissue compartment of the central nervous system (CNS), while human iPSC-derived brain-like microvascular endothelial cells (BMVEC) were grown on the lower side of the membrane to mimic the brain microvasculature (Fig. 2e). This model served as an in vitro platform to test how quantum dots (QDs) engineered with surface functional groups interact with the BBB. A key finding of this study was that in comparison to control particles, Ang-2-coated QDs showed significantly enhanced capacity to move across the BBB into the CNS channel without altering barrier integrity. Importantly, the results of these two representative studies support ongoing efforts to utilize Ang-2-modified drug delivery vehicles for brain disease treatments [80, 81] and also demonstrate how organ-on-a-chip technology may be leveraged in this line of investigation.

#### Investigating the effect of microenvironmental cues on nanoparticle delivery

One of the major challenges associated with using conventional in vitro methods for nanoparticle research is to model nanoparticle delivery in the integrated context of the physiological cellular and tissue microenvironment in vivo. Evidence suggests that biochemical (e.g., spatiotemporal gradients of soluble factors) and physical (e.g., fluid shear stress, tissue stretch) cues produced by the complex native environment can have a profound influence on the behavior of nanoparticles [82–84], but traditional in vitro models largely fail to account for contributions of these important components [24, 25]. To address this problem, attempts have been made to leverage the advanced capability of organ-chips to mimic the complexity of the physiological microenvironment for in vitro modeling and prediction of nanoparticle delivery. Specifically, a considerable amount of work has been conducted to utilize organ-chips to study nanoparticle delivery and uptake under the influence of flow-induced shear stresses.

The work by Samuel et al. provides an example of such studies motivated by the problem that endothelial lining of blood vessels represents a major obstacle to transport of intravascularly delivered drug nanocarriers into target tissues [85]. The researchers utilized a microfluidic device to engineer a vascular endothelium using human umbilical vein endothelial cells (HUVECs) and investigate endothelial uptake of QDs and SiO_2_ nanoparticles at various levels of shear stress (0.5, 1, and 5 dyne/cm^2^). Under static conditions, none of the administered particles entered the endothelium. In the presence of fluid flow, however, significant cellular uptake was observed in a shear-dependent manner and showed the greatest extent of intracellular absorption at 0.5 dyne/cm^2^, underscoring the importance of replicating physiological fluid mechanical forces in nanoparticle studies.

**Fig. 3:**
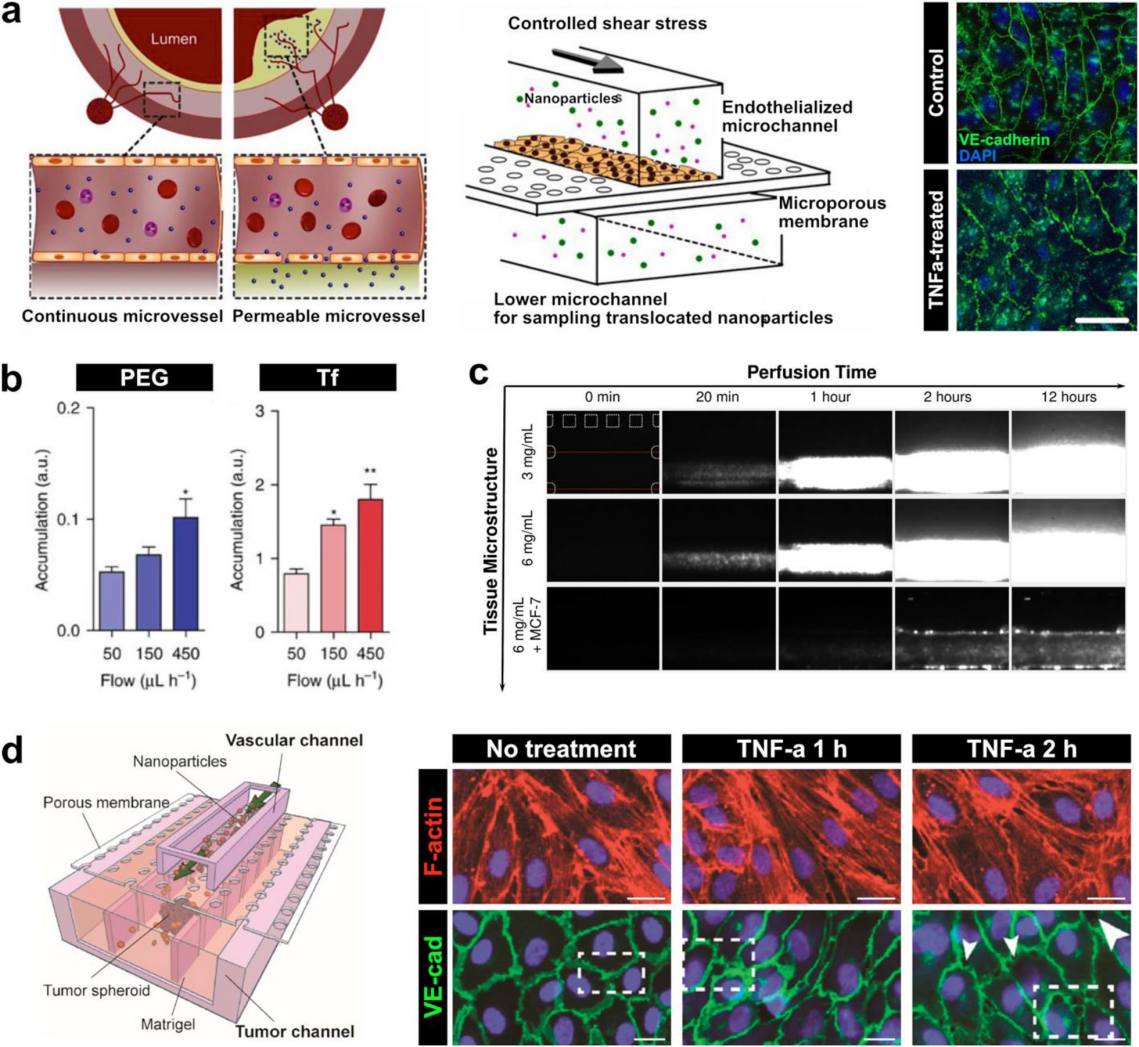
Effect of microenvironmental cues on nanoparticle delivery. **a** An atherosclerosis model to assess the shear and inflammatory effects on membrane permeability. Membrane permeability increases with tumor necrosis factor alpha (TNF-ɑ) treatment and shear stresses, which results in increased nanoparticle translocation. Scale bar, 20 µm. **b** When PEGylated (PEG) and transferrin-functionalized (Tf) nanoparticles were flowed across a tumor-on-a-chip model (Fig. 2b), accumulation in tumor spheroid interstitial spaces doubled at a flow rate of 450 µl/h compared to a lower rate of 50 µl/h. **c** In the breast cancer tumor model (Fig. 2a), higher collagen concentrations did not affect nanoparticle translocation alone, but translocation increased under high collagen and cell packing. **d** A tumor-vasculature-on-a-chip platform was developed to model the effect of leaky vasculature near tumor sites. TNF-ɑ treatment increased intercellular gaps between endothelial cells (white arrows), mimicking leaky vasculature in vitro. Scale bars, 20 µm

A recent study by Kim et al. also demonstrates the utility of organ-chips for modeling how flow affects nanoparticle delivery [86]. This work employed a three-layer microfluidic device capable of generating a perfusable monolayer of HUVECs to study endothelial nanoparticle translocation in the context of atherosclerosis (Fig. 3a). To mimic the inflamed and highly permeable endothelium commonly seen in atherosclerotic plaques, the model was subjected to shear stress of varying magnitude in combination with tumor necrosis factor-α (TNF-α). Application of 1 and 10 dyne/cm^2^ shear stresses, both under the 15 dyne/cm^2^ required for endothelial cells to remain quiescent, resulted in increased vascular permeability, which was accentuated by TNF-α stimulation. Importantly, there was a significant linear correlation between an increase in permeability and increase in nanoparticle translocation across the endothelium, which was corroborated by an atherosclerotic rabbit model.

Organ-chips have also been used to examine the impact of fluid shear stress on nanoparticle delivery in tumor tissues. In the aforementioned study of Albanese et al. (Fig. 2b), the tumor-on-a-chip model was used to quantitatively analyze the accumulation and penetration of PEGylated nanoparticles and transferrin-functionalized (Tf) gold nanoparticles delivered at rates of 50, 150, and 450 μl/h [70]. For both types of nanoparticles, accumulation in the tumor spheroids was greater by more than two-fold when flow rate was at 450 μl/h compared to 50 μl/h (Fig. 3b). Despite this, there were no significant differences in the depth of nanoparticle penetration into the tumor tissues among the different flow rates.

In addition to the investigation of fluid shear stress, recent studies have explored the use of organ-chip platforms to address the question of how complex 3D microarchitecture of in vivo tissues influences the performance of nanotherapeutics. Many of these studies have focused on modeling the tumor microenvironment in which high cell packing densities and increased extracellular matrix (ECM) content of tumor tissues can pose physical barriers to nanoparticle transport [87–89]. For instance, Kwak et al. used the breast tumor-on-a-chip described in the previous section (Fig. 2a) to investigate nanoparticle transport while varying the concentration of collagen and the density of cancer cells in the tumor channel [69]. The data showed that increased collagen content of the tumor constructs had minimal effects on intratumoral delivery of nanoparticles (Fig. 3c). When tumor density was increased by using larger numbers of breast cancer cells to form tumor spheroids, however, nanoparticle penetration and accumulation into the interstitial spaces decreased by approximately 80% (Fig. 3c). These results suggest that physical barriers provided by multicellular architecture should be considered as an important factor during the development of drug nanocarriers for cancer treatment.

Based on the observation of leaky vasculature as one of the characteristics of the tumor microenvironment [90, 91], researchers have also used organ-chip platforms to model this key pathophysiological feature and its implications in nanoparticle transport. Blood vessels with compromised endothelial barrier function, which often form within and in the vicinity of tumors as a result of rapid and disordered tissue growth, have been shown to promote nanoparticle extravasation, accumulation, and retention at tumor tissues. This phenomenon, known as the enhanced permeability and retention (EPR) effect, is one of the leading principles for nanocarrier development [87, 92, 93], which has also motivated recent organ-chip studies. In a representative study by Wang et al., a tumor vasculature-on-a-chip (TVOC) was developed in a three-layer PDMS device by growing HUVECs in the upper channel and 3D tumor spheroids of human ovarian cancer cells (SKOV3) in a central lane of the lower channel (Fig. 3d) [94]. To simulate compromised barrier function of tumor-associated leaky vasculature, HUVECs were treated with TNF-α to induce the formation of intercellular gaps. Exposure to TNF-α for 2 h resulted in significant reductions in cell–cell contacts compared to exposure for 1 h and larger intercellular gap areas similar to those previously observed in vivo. Furthermore, in this altered environment, transport of PEGylated liposomes and poly(lactide-*co*-glycolic acid) (PLGA) nanoparticles across the endothelium increased significantly, highlighting the ability of the TVOC platform to model the leaky vasculature characteristic of the EPR effect.

### Organs-on-a-chip for modeling therapeutic efficacy of drug-carrying nanoparticles

Organ-on-a-chip technology makes it possible to mimic not only normal physiological functions of human tissues and organ units but also disruption of their homeostasis that leads to disease states [61, 95]. Specialized in vitro disease models created in organ-chips hold great potential as new platforms for mechanistic studies of disease pathophysiology, as well as for preclinical assessment of therapeutics [31, 96]. In this section, we provide a survey of how organ-chips have been used for modeling the therapeutic efficacy of nanoparticle-based drug delivery vehicles developed for treatment of cancer and other diseases.

#### Simulating the efficacy of nanotherapeutics for cancer treatment

Despite significant progress in our understanding of cancer pathophysiology, much remains to be accomplished to develop more efficacious and safer cancer therapies [97, 98]. In an attempt to create new in vitro technologies towards this goal, researchers have used approved anticancer nanotherapeutics in current clinical use to demonstrate the potential of cancer-on-a-chip technology for preclinical screening of drug efficacy.

**Fig. 4:**
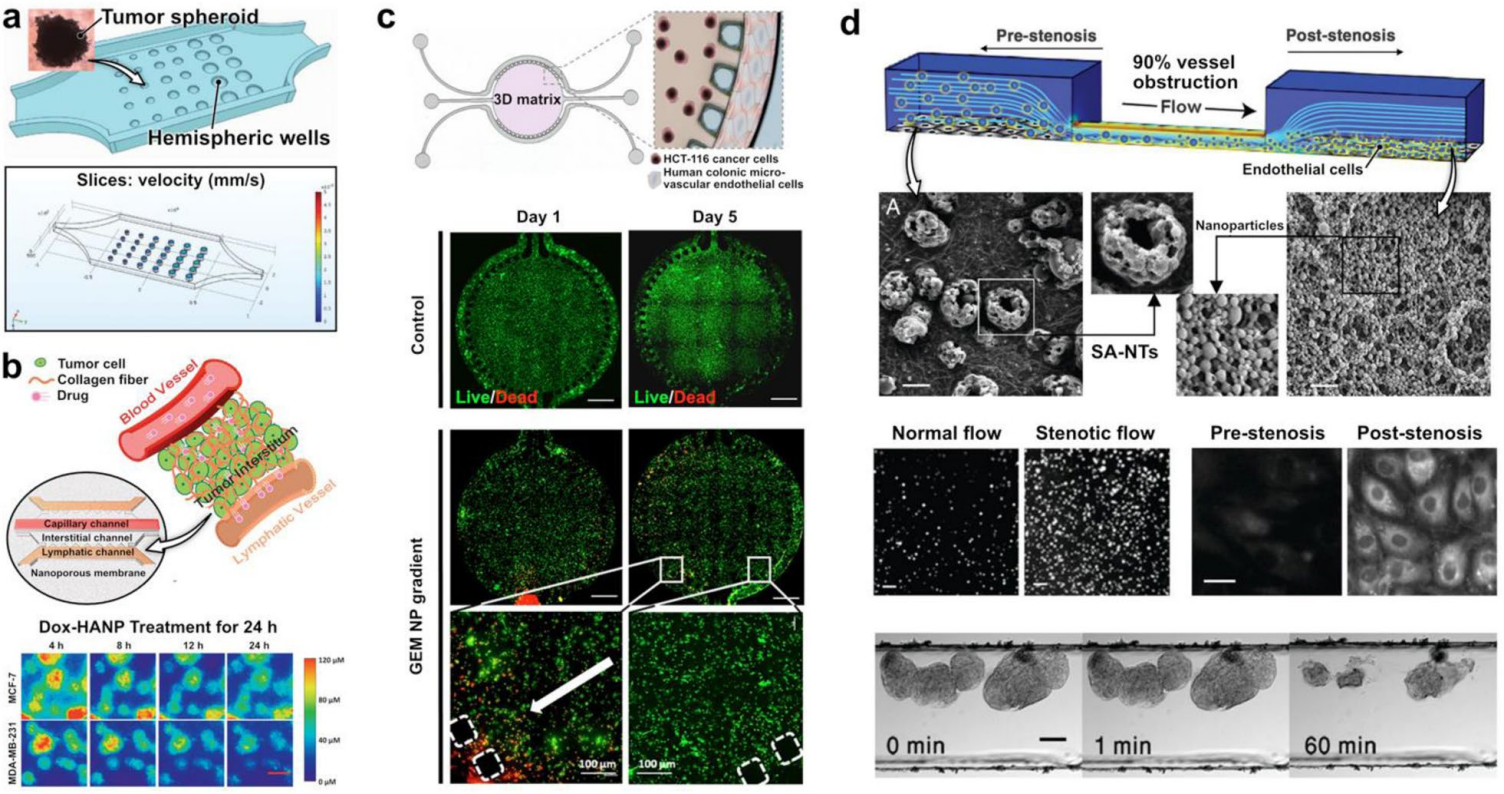
Assessing therapeutic efficacy of nanoparticles. **a** A tumor-on-a-chip model to flow paclitaxel (PTX)-loaded nanoparticles across tumor spheroids held in hemispheric wells. Under flow conditions, the platform demonstrated increased treatment efficacy for high doses and lower flow rates. **b** A tumor-microenvironment-on-a-chip was used to assess the effect of doxorubicin-loaded hyaluronic acid nanoparticles (Dox-HANP). Nanoparticles accumulated more near MCF-7 cells breast cancer cells than MDA-MB-231 breast cancer cells. Scale bar, 50 µm. **c** A human colorectal tumor model was used to culture colon cancer cells in a circular central chamber and endothelial cells in the side channels. Introduction of gemcitabine (GEM)-loaded dendrimer nanoparticles showed a gradient dependent decay of cancer cell viability. Scale bars, 100 µm. **d** Novel shear-activated nanotherapeutics (SA-NTs) were flowed through a microfluidic device modelling a constricted vessel in the study of stenosis. High shear forces resulted in the SA-NTs breaking into individual nanoparticles, resulting in 10 times more nanoparticles after stenotic flow compared to normal flow. Greater accumulation was observed at endothelial cells post-stenosis than pre-stenosis. When SA-NTs were coated with a thrombolytic drug, the individual nanoparticles were able to accumulate at fibrin clots and dissolve them. Scale bars, 2 µm (top), 2 µm (middle left), 20 µm (middle right), 100 µm (bottom)

For example, Ran et al. utilized a tumor-on-a-chip (TOC) to evaluate four liposomes loaded with Paclitaxel (PTX), a drug commonly used for treatment of breast, lung, and ovarian cancer [99]. In this system, liposomes modified with PEG, folic acid (FA), cell penetrating peptide TAT, or both FA and TAT were loaded with 0.1, 0.5, and 1 μg/ml of PTX and administered into a chamber containing an array of human ovarian, SKOV3 tumor spheroids in hemispherical wells at a flow rate of 1 μl/min (Fig. 4a). Drug treatment at low PTX levels of 0.1 μg/ml in this flow system did not produce notable cytotoxic effects, nor did it induce any changes in the size of tumors for any of the modified liposomes. This result was in contrast to significantly decreased cancer cell viability and tumor size observed in static culture of the same type of spheroids at the same PTX concentration, which may be because static cultures can over-amplify cytotoxicity results from their inability to clear nanoparticle sedimentation. However, a higher PTX level of 1 μg/ml in the TOC model demonstrated a significantly lower tumor viability in the TAT and FA-TAT modified liposomes compared to the PEG and FA modified liposomes, which had no difference from the untreated group. The researchers were also able to leverage their platform to study the effects of fluid flow rates (0.25, 1, and 4 μl/min) on tumor tissue inhibition at a constant PTX concentration of 1 μg/ml. At a higher flow rate of 4 μl/min, the tumor spheroid suppression was significantly weaker than at lower flow rates of 0.25 and 1 μl/min, indicating that lower flow rates result in an enhanced treatment efficacy.

Another study demonstrated the capability of a microengineered cancer model to mimic the tumor microenvironment for the study of nanoparticle-mediated anticancer therapy. Shin et al. cultured two human breast cancer lines, MCF-7 and MDA-MB-231, in the cancer-on-a-chip shown in Fig. 2a in order to assess their drug response to doxorubicin (Dox) encapsulated in hyaluronic acid (HA) nanoparticles (Fig. 4b) [100]. In comparison to monolayer culture, cancer cell survival rates were overall greater in the cancer-on-a-chip model, which was attributed most likely to the presence of barriers to nanoparticle transport such as the endothelium and fluid flow that are not present in traditional static culture. Interestingly, the Dox-encapsulating nanoparticles accumulated in MCF-7 cells to a much greater extent than in MDA-MB-231 cells (Fig. 4b), consistent with previous demonstrations of the ability of HA nanoparticles to target cancer cells with the overexpression of CD44 like MCF-7 [101]. It was also noted that there was greater pericellular accumulation of Dox-encapsulating nanoparticles compared to free Dox administered without nanoparticle encapsulation, but the killing of cancer cells was found to be more pronounced with free Dox presumably due to inefficient release of Dox from the nanoparticles.

Lastly, to develop an organ-chip platform for efficacy assessment of anti-colorectal cancer drugs, Carvalho et al. created a compartmentalized microfluidic device consisting of a circular chamber containing Matrigel flanked by two perfusable microchannels (Fig. 4c) [102]. Human colon cancer cells (HCT-116) were embedded in the Matrigel scaffold and co-cultured with human colonic microvascular endothelial cells (HCoMECs) grown into the two side channels. This microengineered cancer construct was used to test the efficacy of gemcitabine (2ʹ,2ʹ-difluoro-2ʹ-deoxycytidine) (GEM), which is a potential treatment option for advanced colorectal cancer currently undergoing clinical trials, loaded into dendrimer nanoparticles. The results of drug testing showed tumor killing effects of GEM-containing nanotherapeutics administered into the vascular channels, which was demonstrated by a significant decrease in the viability of the HCT-116 cells (Fig. 4c). Efficacy of GEM, however, was observed in a spatially graded manner in which the number of dead cancer cells in the hydrogel decreased gradually with increasing distance from the vascular compartment (Fig. 4c), suggesting a gradient of drug-carrying nanoparticles due to their diffusive transport into the ECM scaffold.

#### Modeling the treatment of other diseases

In addition to cancer, microengineered cell culture systems have been used to model other complex diseases for the purposes of assessing the therapeutic potential of nanoparticle drugs. As a representative example, a study by Korin et al. demonstrated the feasibility of using a simple microfluidic culture device to mimic stenotic blood vessels for in vitro testing of novel nanoparticles that can be used for treating acute thrombosis [103]. A PDMS device with varying cross-sectional area was constructed to simulate 90% obstruction of a vessel lumen, which was then seeded with endothelial cells to form a vascular endothelium (Fig. 4d). This study focused on testing microaggregates coated with a thrombolytic drug, tissue plasminogen activator (tPA), that can break up into nanoparticles when exposed to high shear stress. The shear-activated nanotherapeutics (SA-NTs) were found to release 10 times as many nanoparticles when flowing through the constricted region of the device due to increased fluid shear stress, which also led to greater nanoparticle accumulation on the endothelial cells in the downstream of the constriction (Fig. 4d). Importantly, when fibrin clots were generated in the constriction of the device, the SA-NTs introduced into the model dispersed into individual tPA-coated nanoparticles and adhered to the surface of the fibrin emboli to dissolve the clots (Fig. 4d), verifying their therapeutic efficacy.

## Organ-chip models to assess adverse effects of nanotherapeutics

Another critically important goal of preclinical assessment of nanoparticles is to understand their capacity to induce off-target toxicities in humans. To examine the potential of organ-on-a-chip technology for these types of studies, here we highlight a representative body of work that demonstrates the development and application of organ-chips for in vitro modeling and analysis of nanoparticle toxicity. The focus of our discussion is on modeling adverse biological effects of metal oxide nanoparticles, gold nanoparticles, and non-metal nanoparticles.

### Toxicity of metal oxide nanoparticles

Metal oxide nanoparticles such as ZnO and TiO_2_ are used in a wide array of applications ranging from consumer products (e.g., paint, sunscreen) to medical imaging to drug delivery [104, 105]. Although these nanoparticles have been studied extensively, there have been many conflicting results regarding their cytotoxic potential [106–109], making our toxicological understanding of the materials incomplete. To address this problem, researchers are exploring the possibility of using human cell-based organ-chips for more reliable and accurate in vitro prediction of toxic responses to metal oxide nanoparticles.

**Fig. 5:**
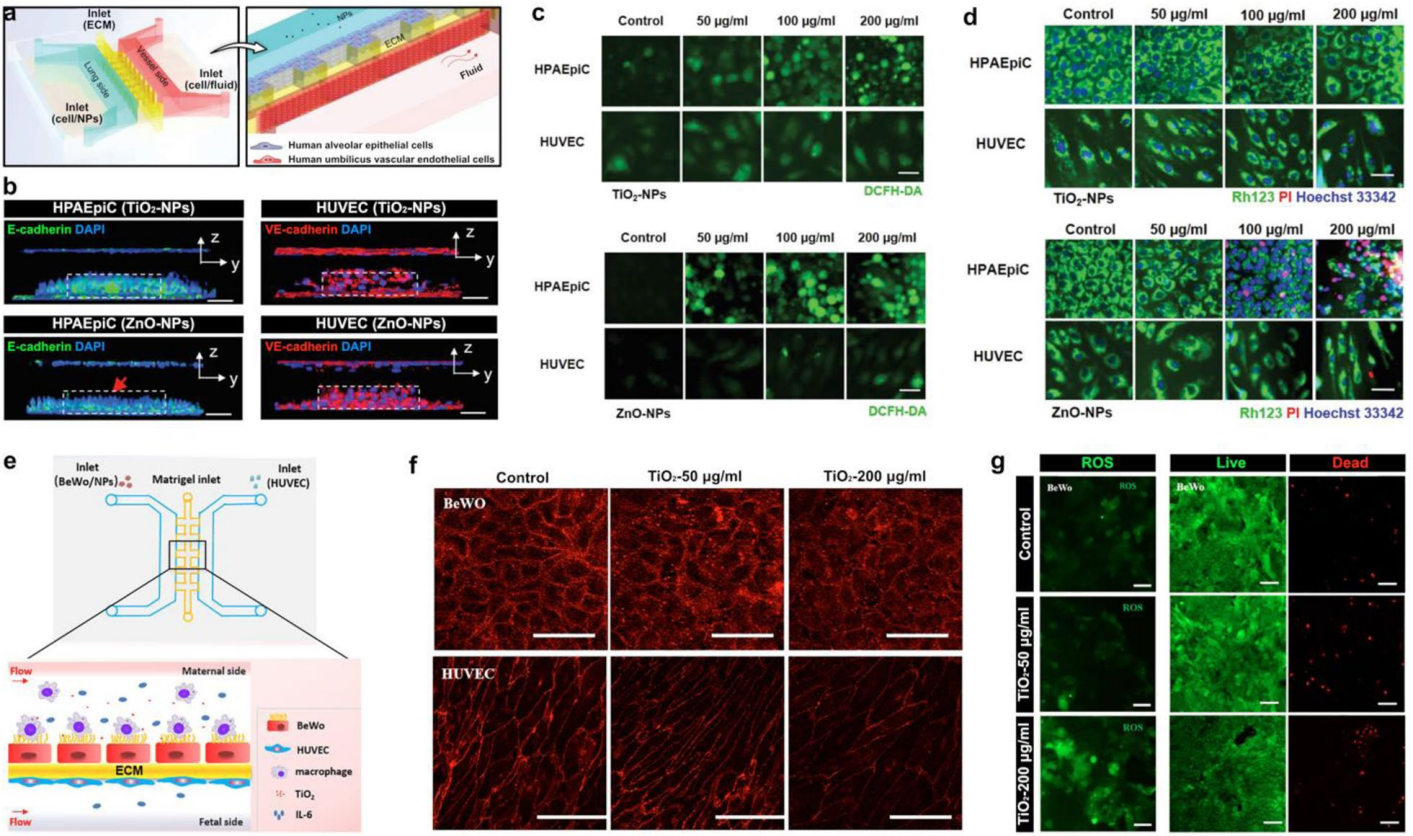
Toxicity of ZnO and TiO_2_ nanoparticles. **a** A lung-on-a-chip model was created by culturing human pulmonary alveolar epithelial cells (HPAEpiC) and human umbilical vein endothelial cells (HUVEC), separated by a Matrigel ECM. **b** Introduction of TiO_2_ nanoparticles did not affect epithelial or endothelial barrers, but ZnO nanoparticles created a noticeable disturbance in the epithelial barrier (red arrow). Scale bars, 100 µm. **c** The reactive oxygen species (ROS) generation and **d** the cellular apoptosis of both nanoparticles was assessed to determine their cytotoxic effects. TiO_2_ nanoparticles induced ROS production in epithelial cells but had no effect on apoptosis. ZnO nanoparticles induced both epithelial and endothelial ROS production and resulted in a dose-dependent increase in epithelial apoptosis. Scale bars, 50 µm. **e** A similar placental barrier-on-a-chip was created using BeWo human trophoblast cells and HUVECs. **f** When TiO_2_ nanoparticles were flown across the device, the placental barrier integrity was compromised. Scale bars, 100 µm. **g** High TiO_2_ concentrations resulted in significant ROS generation and BeWo cell apoptosis. Scale bars, 50 µm (left), 100 µm (middle, right)

For example, Zhang et al. developed a human lung-on-a-chip device to assess the effects of ZnO and TiO_2_ nanoparticles on the alveolar-capillary barrier of the lung by measuring disruption of the barrier, generation of reactive oxygen species (ROS), and cell apoptosis [110]. The device contained three interconnected parallel lanes representing the alveolar and vascular compartments separated by a thin ECM barrier (Fig. 5a). Human tissues in this model were produced by culturing HUVECs and human alveolar epithelial cells (HPAEpiC) in the vessel and lung chambers, respectively, while the ECM compartment in the middle was filled with Matrigel. When TiO_2_ nanoparticles were introduced to the epithelial side of the device to simulate respiratory nanoparticle exposure, the microengineered alveolar-capillary barrier did not exhibit any structural changes but exposure to ZnO nanoparticles resulted in noticeable disruption of barrier integrity (Fig. 5b). In the analysis of ROS, TiO_2_ nanoparticles were found to induce epithelial production of ROS in a dose-dependent manner, whereas oxidative stress was observed in both the epithelial and endothelial populations when the model was treated with ZnO nanoparticles (Fig. 5c). Measurement also revealed that TiO_2_ particles had no significant impact on the apoptosis of the exposed cells, which was in contrast to a dose-dependent increase in epithelial apoptosis as a result of ZnO nanoparticle exposure (Fig. 5d). These results indicating greater overall toxicity of ZnO nanoparticles were in good agreement with previous findings [111, 112], supporting the potential of the lung-on-a-chip system for nanoparticle toxicity screening.

In another study conducted by Yin et al., the toxicity of TiO_2_ nanoparticles was investigated in the context of pregnancy using a microengineered 3D placental barrier-on-a-chip model [113]. Similar to the design of the lung-on-a-chip system described above, a three-lane microdevice was utilized in this model for co-culture of BeWo human trophoblast cells and HUVECs on either side of a Matrigel barrier to mimic the structural organization of the placental barrier in vivo (Fig. 5e). TiO_2_ nanoparticles introduced into the BeWo cell-containing maternal chamber compromised the integrity of the placental barrier as evidenced by reduced expression of cell–cell junctions in both the trophoblast and HUVEC monolayers (Fig. 5f). Furthermore, high concentrations (200 μg/ml) of nanoparticles induced oxidative stress and increased cell death in the BeWo cells (Fig. 5g), illustrating potentially detrimental effects of TiO_2_ nanoparticles on the human placental barrier.

### Toxicity of gold nanoparticles

The ease of synthesis and chemical modification makes gold nanoparticles highly attractive for a wide variety of biomedical applications [114]. While gold nanoparticles are generally considered safe, studies have reported conflicting outcomes, some of which suggest their potential to induce oxidative stress and other deleterious responses [115, 116]. Similar results have been described in a recent study by Fede et al. that used HUVECs grown in a simple microfluidic channel to study how fluid flow affects endothelial responses to gold nanoparticles [117]. The key finding of this study was that toxic responses of HUVECs, which was analyzed by cell viability, was significantly reduced under flow conditions compared to static culture. The data also showed that this protective effect occurred in a dose-dependent manner and was observed at higher nanoparticle concentrations (5 × 10^11^ nanoparticles/ml). This trend was again seen in a later study conducted by the same research group [118], highlighting the importance of recapitulating physiologically relevant microenvironmental cues for in vitro toxicological assessment of gold nanoparticles.

### Toxicity of nonmetal nanoparticles

Nonmetal nanoparticles such as those composed of silica have gained great attention due to their desirable properties, such as easily adjustable surface chemistry and porosity, which also make them appealing for therapeutic applications [104]. Although these particles were originally thought to be highly biocompatible, recent studies have revealed that they have the potential to induce ROS generation [119]. Other nonmetal nanoparticles such as polymeric nanoparticles are also of great interest for the development of nanotherapeutics [120], but work is required to better understand their capacity to exert adverse biological and physiological effects. As is the case with other types of nanoparticles, organ-chips have proven instrumental for these types of studies.

**Fig. 6:**
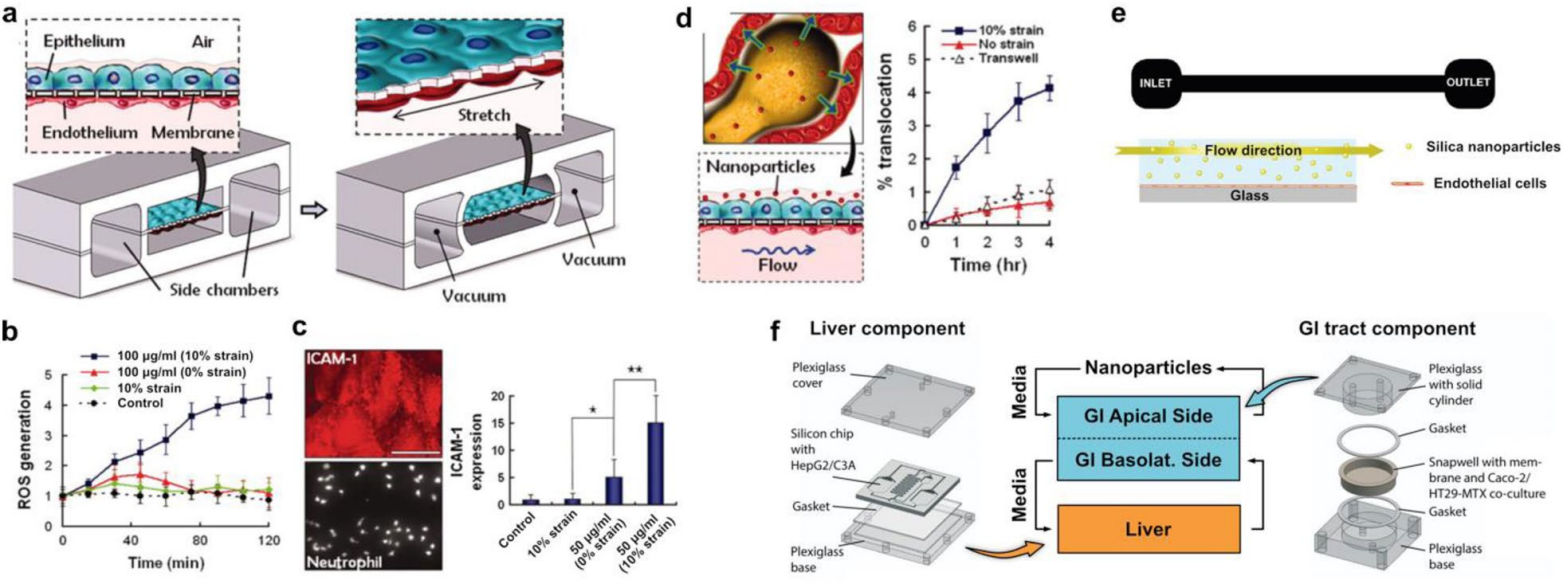
Toxicity of nonmetal nanoparticles. **a** A human breathing lung-on-a-chip was created by culturing human alveolar epithelial cells and pulmonary microvascular endothelial cells on opposite sides of a porous membrane. Vacuum chambers allowed for physiological mechanical breathing stresses to be applied. **b** Introduction of silica nanoparticles in the presence of mechanical cues resulted in significant oxidative stress responses. **c** Mechanical strain also significantly increased the expression of ICAM-1 and neutrophil adhesion to the endothelial side of the device, Scale bar, 50 µm, **d** and increased the translocation of nanoparticles from the alveolar to vascular compartments. **e** A microfluidic device coated with endothelial cells was used to assess the toxicity of silica particles. Mechanical stimulation through shear stresses resulted in lower cell viabilities. **f** A liver and gastrointestinal system body-on-a-chip was created to study the liver injury potential of carboxylated polystyrene nanoparticles. Introduction of the nanoparticles to the apical side of the gastrointestinal (GI) component resulted in a dose-dependent increase in enzymes indicating liver injury

For example, Huh et al. used a biomimetic human breathing lung on-a-chip model of the alveolar-capillary interface to study adverse respiratory effects of silica nanoparticles [36]. This system was constructed by growing human alveolar epithelial cells and pulmonary microvascular endothelial cells on the opposite sides of a porous flexible membrane (Fig. 6a). A unique design feature of this system was that vacuum was applied to two hollow chambers adjacent to the cell culture channels to induce cyclic stretching of the microengineered bi-layer tissue akin to the deformation of the alveolar-capillary barrier during breathing (Fig. 6a). Importantly, this study demonstrated significant oxidative stress responses of the alveolar epithelium to silica nanoparticles in the presence of physiological breathing-induced mechanical forces (Fig. 6b). Deleterious effects of silica nanoparticles were also shown by the activation of the vascular endothelium, which was accentuated by the application of breathing-induce forces (Fig. 6c). Moreover, mechanical strain significantly increased the rate of silica nanoparticle translocation from the alveolar compartment to the vasculature (Fig. 6d), which was corroborated by increased extrapulmonary absorption in an ex vivo mouse lung ventilation–perfusion model. Together, these results showed that breathing motions may exacerbate the toxic effects of respiratory exposure to silica nanoparticles.

The significance of physiological biomechanical forces for in vitro toxicity assessment of silica nanoparticles was also demonstrated in a study conducted by Kim et al. [121]. The authors cultured human endothelial cells in a simple flow-through microfluidic system to investigate the effect of fluid shear stress on endothelial responses to the silica particles (Fig. 6e). Similar to the work of Huh et al., mechanical stimulation of the endothelium with fluid flow in this model resulted in significantly decreased cell viability compared to static control.

Lastly, researchers have recently investigated the safety of other nonmetal nanoparticles, such as carboxylated polystyrene nanoparticles, in organ-chip models. To determine the potential of these nanoparticles to induce liver injury, Esch et al. developed a body-on-a-chip device consisting of intestinal and liver models that were fluidically linked together [56]. Specifically, Caco-2 intestinal epithelial cells and mucin-producing TH29-MTX cells were cocultured in a microfluidic chamber representing the intestine, which was connected to another microfabricated chamber containing HepG2/C3A liver cells (Fig. 6f). When carboxylated polystyrene nanoparticles were introduced into the apical side of the intestinal model, the HepG2/C3A cells showed a dose-dependent increase in the production of tissue-specific enzymes indicative of liver injury. Importantly, this integrated multi-organ model showed a greater extent of nanoparticle-induced livery injury as a result of interactions between the liver and the intestinal epithelium.

## Key challenges and future opportunities

In the past few decades, the potential of engineered nanoparticles for therapeutic applications has become increasingly apparent. There is growing recognition that the limitations of existing in vitro and animal models are among the roadblocks to translating preclinical findings in nanoparticle research into efficacious and safe therapeutics for practical clinical use. Despite its infancy, the idea of harnessing the power of organs-on-a chip to overcome these limitations have gained much traction in recent years. To advance the potential of this disruptive technology for nanomedicine, efforts should be made in future studies to address the following challenges.

While considerable progress has been made in the organ-on-a-chip field, work remains to be done to enhance our ability to emulate the complexity of how human physiological systems respond to nanoparticles. One of the key challenges has been to recapitulate the interaction of engineered nanoparticles with the immune system, which is believed to have a profound influence on virtually every aspect of nanoparticle delivery, efficacy, and toxicity [122]. As illustrated by recent development of lymph nodes-on-a-chip [123, 124], mimicking immune organs is emerging as an area of active research investigation in the organ-chip field. Future studies should explore the integration of these new systems with target organ models, which may enable more accurate and reliable predictions of transport and biological function of nanotherapeutics.

As is the case with other applications of organ-on-a-chip technology, it remains a great challenge to validate the physiological and clinical relevance of in vitro findings made by organ-chip models for nanoparticle research. Ironically, the most common approach to model validation has been to conduct in vitro-in vivo correlations using data generated by animal models whose limitations have motivated the development of human cell-based organ-chip systems to begin with [70, 86]. Addressing this problem will likely require the formation of new alliances and partnerships between organ-chip developers, clinical toxicologists, and pharmaceutical companies to maximize the availability of human clinical data that can be used for comparative analysis during model validation. Such data will also inform the process of designing organ chip models, as well as their biological and physiological endpoints that need to be analyzed.

In typical research settings, most organ-chip systems are constructed using PDMS due to its low cost, optical transparency, biocompatibility, gas permeability, and ease of fabrication. However, a major drawback of PDMS is its absorption of small hydrophobic molecules [125, 126], which can be particularly problematic for drug testing applications. While the use of nanocarriers is presumed to be advantageous for preventing this issue, questions remain whether and how nanotherapeutics containing hydrophobic payloads or surface groups interact with PDMS, especially given a wide variety of surface engineering techniques exploited for developing functional nanoparticles. Future studies should treat this as an important issue and systematically investigate the effect of PDMS and other synthetic materials used in organ-chips (e.g., semipermeable membranes) on the delivery and performance of nanoparticles. As another mitigation strategy, attention should also be paid to ongoing efforts to develop alternative materials to PDMS [127].

With these challenges also come new opportunities. With rapid advancement of organoid technology [128–130], new efforts are being made to incorporate organoids and other self-organizing biological structures into the precisely controlled microenvironment of organ-chips [131, 132]. It is clear that nanoparticle research will benefit from these types of systems that offer more sophisticated, realistic, and predictive platforms for preclinical studies. On a related note, future studies should also take advantage of considerable progress in the development of self-assembled, perfusable 3D human blood vessels in microdevices [133–135]. Integrating the engineered perfusable vasculature into microphysiological systems will make it possible to emulate delivery and bioactivity of nanotherapeutics in a more physiologically relevant manner, allowing for accurate analysis and prediction of their performance in vivo.

Recent studies have also met with great success in demonstrating the feasibility of creating microengineered in vitro models using iPSCs as a sustainable source of primary human cells [58, 59]. iPSC-based organ-chips have attracted great attention because they enable the production of preclinical models that can replicate disease phenotypes in a patient-specific manner [136, 137]. These kinds of systems may provide a potentially powerful platform to develop personalized nanotherapeutics and drug delivery technologies tailored specifically to the conditions and needs of patients.

Another promising research direction is to advance analytical capabilities of existing organ-chips by integrating sensing components into microfluidic cell culture. While the development of such integrated models may present non-trivial design and engineering challenges, this approach may expand our ability to interrogate how microengineered tissue and organ units respond to nanotherapeutics. In particular, electrochemical and optical sensors capable of real-time measurement of morphological and biochemical changes will be instrumental for spatiotemporal analysis of biological responses occurring over short time scales that may not be easily resolved using conventional techniques. In a similar vein, efforts should also be made to explore how advances in single cell sequencing and bioinformatics technologies can be leveraged to gain in-depth, mechanistic understanding of biological responses to nanoparticles in the physiological context of organ-chip models.

Finally, creating body-on-a-chip models of nanoparticle efficacy and toxicity represents an exciting opportunity for future research of nanomedicine. Improving our incomplete understanding of the behavior of nanoparticles in the body will involve addressing the question of how nanoparticles administered into the systemic circulation interact with multiple physiological systems that are structurally and functionally associated with target organs. Filling this knowledge gap in the current body of work will require the development of integrated model systems that combine multiple interacting cell culture units representing different organs, as well as in vitro techniques and culture protocols optimized to meet complex media conditions of multiple cell and tissue types. Given that body-on-a-chip systems have been used with great success for in vitro assessment of drug toxicities [138], future work should explore the possibility of extending similar approaches to the study of therapeutic nanoparticles.

## Conclusion

With the advancement of organ-on-a-chip technology, new opportunities are emerging to harness its novel capabilities and potential to disrupt the process of developing nanomedicine. Microengineered biomimetic models of human tissues and functional organ units can provide more physiologically relevant and predictive in vitro platforms to examine responses of human physiological systems to nanomaterials in ways that have not been possible using traditional cell culture techniques. As highlighted in this review, the convergence of this technology with nanomedicine offers the promise of more accurate and reliable preclinical assessment of engineered nanoparticles for therapeutic applications. Work directed towards fulfilling this promise is in its infancy, and significant advances have yet to be made in tailoring the design and capabilities of organs-on-a-chip to the specific needs in the field of nanomedicine. With rapidly increasing research interests in this area, however, it is not difficult to foresee that organ-on-a-chip technology will play an instrumental role in the development of nanotherapeutics in the future. As enabling in vitro platforms with unprecedented predictive capacity, organs-on-a-chip are well-poised to make great contributions to advancing the frontier of nanomedicine.

## Data Availability

Any data related to this review are available from the corresponding author on reasonable request.

## Abbreviations

AIDS: Acquired immunodeficiency syndrome
3D: Three-dimensional
MPS: Microphysiological systems
PDMS: Poly(dimethylsiloxane)
RBE4: Rat brain endothelial cells
ICAM-mAb: Anti-intracellular adhesion molecule-1 antibody
IgG: Immunoglobulin G antibody
BBB: Blood brain barrier
Ang-2: Angiopep-2
CNS: Central nervous system
iPSC: Induced pluripotent stem cells
QD: Quantum dot
HUVEC: Human umbilical vein endothelial cells
TNF-α: Tumor necrosis factor-α
ECM: Extracellular matrix
EPR: Enhanced permeability and retention
TVOC: Tumor vasculature-on-a-chip
PLGA: Poly(lactide-*co*-glycolic acid)
TOC: Tumor-on-a-chip
PTX: Paclitaxel
FA: Folic acid
Dox: Doxorubicin
HA: Hyaluronic acid
GEM: Gemcitabine (2ʹ, 2ʹ-difluoro-2ʹ-deoxycytidine)
tPA: Tissue plasminogen activator
SA-NT: Shear-activated nanotherapeutics

## References

[1] Gao X, Cui Y, Levenson RM, Chung LW, Nie S (2004). Nat. Biotechnol..

[2] Kim S, Lim YT, Soltesz EG, De Grand AM, Lee J, Nakayama A, Parker JA, Mihaljevic T, Laurence RG, Dor DM, Cohn LH, Bawendi MG, Frangioni JV (2004). Nat. Biotechnol..

[3] Harisinghani MG, Barentsz J, Hahn PF, Deserno WM, Tabatabaei S, van de Kaa CH, de la Rosette J, Weissleder R (2003). NEJM.

[4] Sandhu A, Handa H, Abe M (2010). Nanotechnology.

[5] Brigger I, Dubernet C, Couvreur P (2012). Adv. Drug Deliv. Rev..

[6] Sengupta S, Eavarone D, Capila I, Zhao G, Watson N, Kiziltepe T, Sasisekharan R (2005). Nature.

[7] Heo DN, Yang DH, Moon HJ, Lee JB, Bae MS, Lee SC, Lee WJ, Sun IC, Kwon IK (2012). Biomaterials.

[8] Schneider CS, Xu Q, Boylan NJ, Chisholm J, Tang BC, Schuster BS, Henning A, Ensign LM, Lee E, Adstamongkonkul P, Simons BW, Wang SS, Gong X, Yu T, Boyle MP, Suk JS, Hanes J (2017). Sci. Adv..

[9] Wang W, Zhu R, Xie Q, Li A, Xiao Y, Li K, Liu H, Cui D, Chen Y, Wang S (2012). Int. J. Nanomed..

[10] Mastorakos P, da Silva AL, Chisholm J, Song E, Choi WK, Boyle MP, Morales MM, Hanes J, Suk JS (2015). PNAS.

[11] Wilson B, Samanta MK, Santhi K, Kumar KPS, Paramakrishnan N, Suresh B (2008). Brain Res..

[12] Joshi SA, Chavhan SS, Sawant KK (2010). Eur. J. Pharm. Biopharm..

[13] Lobenberg R, Araujo L, von Briesen H, Rodgers E, Kreuter J (1998). J. Control. Release.

[14] Shah LK, Amiji MM (2006). Pharm. Res..

[15] Lewis DR, Petersen LK, York AW, Zablocki KR, Joseph LB, Kholodovych V, Prud’homme RK, Uhrich KE, Moghe PV (2015). PNAS.

[16] Kim YI, Flukiger L, Hoffman M, Lartaud-Idjouadiene I, Atkinson J, Maincent T (1997). Br. J. Pharmacol..

[17] Ludwig A (2005). Adv. Drug Deliv. Rev..

[18] Pignatello R, Bucolo C, Spedalieri G, Maltese A, Puglisi G (2002). Biomaterials.

[19] Pignatello R, Bucolo C, Ferrara P, Maltese A, Puleo A, Puglisi G (2002). Eur. J. Pharm. Sci..

[20] Ventola CL (2012). Pharm. Ther..

[21] Khan I, Saeed K, Khan I (2019). Arab. J. Chem..

[22] Patra JK, Das G, Fraceto LF, Campos EVR, del Pilar Rodriguez-Torres M, Acosta-Torres LS, Diaz-Torres LA, Grillo R, Swamy MK, Sharma S, Habtemariam S, Shin HS (2018). J. Nanobiotechnol..

[23] Rizvi SA, Saleh AM (2018). Saudi Pharm. J..

[24] Mitchell MJ, Billingsley MM, Haley RM, Wechsler ME, Peppas NA, Langer R (2021). Nat. Rev. Drug Discov..

[25] Hua S, De Matos MB, Metselaar JM, Storm G (2018). Front. Pharmacol..

[26] Anselmo AC, Mitragotri S (2019). Bioeng. Transl. Med..

[27] Stirland DL, Nichols JW, Miura S, Bae YH (2013). J. Control. Release.

[28] Van Norman GA (2019). JACC Basic Transl. Sci..

[29] Huh D, Hamilton GA, Ingber DE (2011). Trends Cell Biol..

[30] Bhatia SN, Ingber DE (2014). Nat. Biotechnol..

[31] Esch EW, Bahinski A, Huh D (2015). Nat. Rev. Drug Discov..

[32] Zhang B, Korolj A, Lai BFL, Radisic M (2018). Nat. Rev. Mater..

[33] Huh D, Torisawa YS, Hamilton GA, Kim HJ, Ingber DE (2012). Lab Chip.

[34] Park SE, Georgescu A, Huh D (2019). Science.

[35] Seo J, Byun WY, Alisafaei F, Georgescu A, Yi YS, Massaro-Giordano M, Shenoy VB, Lee V, Bunya VY, Huh D (2019). Nat. Med..

[36] Huh D, Matthews BD, Mammoto A, Montoya-Zavala M, Hsin HY, Ingber DE (2010). Science.

[37] Stucki AO, Stucki JD, Hall SR, Felder M, Mermoud Y, Schmid RA, Geiser T, Guenat OT (2015). Lab Chip.

[38] Kim HJ, Li H, Collins JJ, Ingber DE (2016). PNAS.

[39] Bein A, Shin W, Jalili-Firoozinezhad S, Park MH, Sontheimer-Phelps A, Tovaglieri A, Chalkiadaki A, Kim HJ, Ingber DE (2018). Cell. Mol. Gastroenterol. Hepatol..

[40] Bhise NS, Manoharan V, Massa S, Tamayol A, Ghaderi M, Miscuglio M, Lang Q, Zhang YS, Shin SR, Calzone G, Annabi N, Shupe TD, Bishop CE, Atala A, Dokmeci MR, Khademhosseini A (2016). Biofabrication.

[41] Lee KH, Lee J, Lee SH (2015). Lab Chip.

[42] Wilmer MJ, Ng CP, Lanz HL, Vulto P, Suter-Dick L, Masereeuw R (2016). Trends Biotechnol..

[43] Jang KJ, Mehr AP, Hamilton GA, McPartlin LA, Chung S, Suh KY, Ingber DE (2013). Integr. Biol..

[44] Deosarkar SP, Prabhakarpandian B, Wang B, Sheffield JB, Krynska B, Kiani MF (2015). PLoS ONE.

[45] Kilic O, Pamies D, Lavell E, Schiapparelli P, Feng Y, Hartung T, Bal-Price A, Hogberg HT, Hinojosa AQ, Cazares HG, Levchenko A (2016). Lab Chip.

[46] Lee JS, Romero R, Han YM, Kim HC, Kim CJ, Hong JS, Huh D (2016). J. Matern. Fetal Neonatal Med..

[47] Blundell C, Yi YS, Ma L, Tess ER, Farrell ER, Georgescu A, Aleksunes LM, Huh D (2018). Adv. Healthc. Mater..

[48] Xia Y, Whitesides GM (1998). Annu. Rev. Mater. Sci..

[49] Whitesides GM, Ostuni E, Takayama S, Jiang X, Ingber DE (2001). Annu. Rev. Biomed. Eng..

[50] Qin D, Xia Y, Whitesides GM (2010). Nat. Protoc..

[51] Sung JH, Wang YI, Narasimhan Sriram N, Jackson M, Long C, Hickman JJ, Shuler ML (2018). Anal. Chem..

[52] Yesil-Celiktas O, Hassan S, Miri AK, Maharjan S, Al-kharboosh R, Quinones-Hinojosa A, Zhang YS (2018). Adv. Biosyst..

[53] Tang H, Abouleila Y, Si L, Ortega-Prieto AM, Mummery CL, Ingber DE, Mashaghi A (2020). Trends Microbiol..

[54] Kimura H, Sakai Y, Fujii T (2018). Drug Metab. Pharmacokinet..

[55] Ronaldson-Bouchard K, Vunjak-Novakovic G (2018). Cell Stem Cell.

[56] Esch MB, Mahler GJ, Stokol T, Shuler ML (2014). Lab Chip.

[57] Maschmeyer I, Lorenz AK, Schimek K, Hasenberg T, Ramme AP, Hubner J, Lindner M, Drewell C, Bauer S, Thomas A, Sambo NS, Sonntag F, Lauster R, Marx U (2015). Lab Chip.

[58] Sances S, Ho R, Vatine G, West D, Laperle A, Meyer A, Godoy M, Kay PS, Mandefro B, Hatata S, Hinojosa C, Wen N, Sareen D, Hamilton GA, Svendsen CN (2018). Stem Cell Rep..

[59] Vatine GD, Barrile R, Workman MJ, Sances S, Barringa BK, Rahnama M, Barthakur S, Kasendra M, Lucchesi C, Kerns J, Wen N, Spivia WR, Chen Z, Eyk JV, Svendsen CN (2019). Cell Stem Cell.

[60] Abulaiti M, Yalikum Y, Murata K, Sato A, Sami MM, Sasaki Y, Fujiwara Y, Minatoya K, Shiba Y, Tanaka Y, Masumoto H (2020). Sci. Rep..

[61] Skardal A, Shupe T, Atala A (2016). Drug Discov. Today.

[62] Takebe T, Zhang B, Radisic M (2017). Cell Stem Cell.

[63] Low LA, Mummery C, Berridge BR, Austin CP, Tagle DA (2020). Nat. Rev. Drug Discov..

[64] Wu Q, Liu J, Wang X, Feng L, Wu J, Zhu X, Wen W, Gong X (2020). Biomed. Eng. Online.

[65] Friedman AD, Claypool SE, Liu R (2013). Curr. Pharm. Des..

[66] De Jong WH, Borm PJ (2008). Int. J. Nanomed..

[67] Albanese A, Tang PS, Chan WC (2012). Annu. Rev. Biomed. Eng..

[68] Banerjee A, Qi J, Gogoi R, Wong J, Mitragotri S (2016). J. Control. Release.

[69] Kwak B, Ozcelikkale A, Shin CS, Park K, Han B (2014). J. Control. Release.

[70] Albanese A, Lam AK, Sykes EA, Rocheleau JV, Chan WC (2013). Nat. Commun..

[71] Toy R, Peiris PM, Ghaghada KB, Karathanasis E (2014). Nanomedicine.

[72] Bhise NS, Ribas J, Manoharan V, Zhang YS, Polini A, Massa S, Dokmeci MR, Khademhosseini A (2014). J. Control. Release.

[73] Kolhar P, Anselmo AC, Gupta V, Pant K, Prabhakarpandian B, Ruoslahti E, Mitragotri S (2013). PNAS.

[74] Nasir I, Lundqvist M, Cabaleiro-Lago C (2015). Nanoscale.

[75] Baer DR, Engelhard MH, Johnson GE, Laskin J, Lai J, Mueller K, Munusamy P, Thevuthasan S, Wang H, Washton N, Elder A, Baisch BL, Karakoti A, Kuchibhatla SVNT, Moon D (2013). J. Vac. Sci. Technol. A Vac. Surf. Films..

[76] Papademetriou I, Vedula E, Charest J, Porter T (2018). PLoS ONE.

[77] Demeule M, Currie JC, Bertrand Y, Che C, Nguyen T, Regina A, Gabathuler R, Castaigne JP, Beliveau R (2008). J. Neurochem..

[78] Demeule M, Regina A, Che C, Poirier J, Nguyen T, Gabathuler R, Castaigne JP, Beliveau R (2008). J. Pharmacol. Exp. Ther..

[79] Park TE, Mustafaoglu N, Herland A, Hasselkus R, Mannix R, FitzGerald EA, Baun RP, Watters A, Henry O, Benz M, Sanchez H, McCrea HJ, Goumnerova LC, Song HW, Palecek SP, Shusta E, Ingber DE (2019). Nat. Commun..

[80] Papademetriou IT, Porter T (2015). Ther. Deliv..

[81] Huile G, Shuaiqi P, Zhi Y, Shijie C, Chen C, Xinguo J, Shun S, Zhiqing P, Yu H (2011). Biomaterials.

[82] Klingberg H, Loft S, Oddershede LB, Moller P (2015). Nanoscale.

[83] Hussain S, Garantziotis S, Rodrigues-Lima F, Dupret JM, Baeza-Squiban A, Boland S (2014). Nanomaterial.

[84] Huai Y, Hossen MN, Wilhelm S, Bhattacharya R, Mukherjee P (2019). Bioconjug. Chem..

[85] Samuel SP, Jain N, O’Dowd F, Paul T, Kashanin D, Gerard VA, Gun’ko YK, Mello AP, Volkov Y (2012). Int. J. Nanomed..

[86] Kim Y, Lobatto ME, Kawahara T, Chung BL, Mieszawska AJ, Sanchez-Gaytan BL, Fay F, Senders ML, Calcagno C, Becraft J, Saung MT, Gordon RE, Stroes ESG, Ma M, Farokhzad OC, Fayad ZA, Mulder WJM, Langer R (2014). PNAS.

[87] Fang J, Nakamura H, Maeda H (2011). Adv. Drug Deliv. Rev..

[88] Netti PA, Berk DA, Swartz MA, Grodzinsky AJ, Jain RK (2000). Cancer Res.

[89] Brown E, McKee T, Pluen A, Seed B, Boucher Y, Jain RK (2003). Nat. Med..

[90] Hashizume H, Baluk P, Morikawa S, McLean JW, Thurston G, Roberge S, Jain RK, McDonald DM (2000). Am. J. Pathol..

[91] Azzi S, Hebda JK, Gavard J (2013). Front. Oncol..

[92] Kobayashi H, Watanabe R, Choyke PL (2014). Theranostics.

[93] Golombek SK, May JN, Theek B, Appold L, Drude N, Kiessling F, Lammers T (2018). Adv. Drug Deliv. Rev..

[94] Wang HF, Ran R, Liu Y, Hui Y, Zeng B, Chen D, Weitz DA, Zhao CX (2018). ACS Nano.

[95] Huh D, Leslie DC, Matthews BD, Fraser JP, Jurek S, Hamilton GA, Thorneloe KS, McAlexander MA, Ingber DE (2012). Sci. Transl. Med..

[96] Ingber DE (2016). Cell.

[97] Hare JI, Lammers T, Ashford MB, Puri S, Storm G, Barry ST (2017). Adv. Drug Deliv. Rev..

[98] Rosenblum D, Joshi N, Tao W, Karp JM, Peer D (2018). Nat. Commun..

[99] Ran R, Wang HF, Hou F, Liu Y, Hui Y, Petrovsky N, Zhang F, Zhao CX (2019). Adv. Healthc. Mater..

[100] Shin K, Klosterhoff BS, Han B (2016). Mol. Pharm..

[101] Choi KY, Chung H, Min KH, Yoon HY, Kim K, Park JH, Kwon IC, Jeong SY (2010). Biomaterials.

[102] Carvalho MR, Barata D, Teixeira LM, Giselbrecht S, Reis RL, Oliveira JM, Truckenmuller R, Habibovic P (2019). Sci. Adv..

[103] Korin N, Kanapathipillai M, Matthews BD, Crescente M, Brill A, Mammoto T, Ghosh K, Jurek S, Bencherif SA, Bhatta D, Coskun AU, Feldman CL, Wagner DD, Ingber DE (2012). Science.

[104] Sengul AB, Asmatulu E (2020). Environ. Chem. Lett..

[105] Stark WJ, Stoessel PR, Wohlleben W, Hafner AJCSR (2015). Chem. Soc. Rev..

[106] Ammendolia MG, Losi F, Maranghi F, Tassinari R, Cubadda F, Aureli F, Raggi A, Superti F, Mantovani A, De Berardis B (2017). Food Chem. Toxicol..

[107] Di Bucchianico S, Cappellinim F, Le Bihanic F, Zhang Y, Dreij K, Karlsson HL (2017). Mutagenesis.

[108] Rizk MZ, Ali SA, Hamed MA, El-Rigal NS, Aly HF, Salah HH (2017). Biomed. Pharmacother..

[109] Shi Y, Wang F, He J, Yadav S, Wang H (2010). Toxicol. Lett..

[110] Zhang M, Xu C, Jiang L, Qin J (2018). Toxicol. Res..

[111] Park S, Lee YK, Jung M, Kim KH, Chung N, Ahn EK, Lim Y, Lee KH (2007). Inhal. Toxicol..

[112] Vimercati L, Cavone D, Caputi A, De Maria L, Tria M, Prato E, Ferri GM (2020). Front. Public Health.

[113] Yin F, Zhu Y, Zhang M, Yu H, Chen W, Qin J (2019). Toxicol. In Vitro.

[114] Jia YP, Ma BY, Wei XW, Qian ZY (2017). Chin. Chem. Lett..

[115] Li JJ, Hartono D, Ong CN, Bay BH, Yung LYL (2010). Biomaterials.

[116] Lopez-Chaves C, Soto-Alvaredo J, Montes-Bayon M, Bettmer J, Lopis J, Sanchez-Gonzalez C (2018). Nanomed. Nanotechnol. Biol. Med..

[117] Fede C, Fortunati I, Weber V, Rossetto N, Bertasi F, Petrelli L, Guidolin D, Signorini R, De Caro R, Albertin G, Ferrante C (2015). Microvasc. Res..

[118] Fede C, Albertin G, Petrelli L, De Caro R, Fortunati I, Weber V, Ferrante C (2017). J. Nanopart. Res..

[119] Duan J, Yu Y, Li Y, Yu Y, Li Y, Zhou X, Huang P, Sun Z (2013). PLoS ONE.

[120] Li B, Li Q, Mo J, Dai H (2017). Front. Pharmacol..

[121] Kim D, Lin YS, Haynes CL (2011). Anal. Chem..

[122] Shreffler JW, Pullan JE, Dailey KM, Mallik S, Brooks AE (2019). Int. J. Mol. Sci..

[123] Shanti A, Samara B, Abdullah A, Hallfors N, Accoto D, Sapudom J, Alatoom A, Teo J, Danti S, Stefanini C (2020). Pharmaceutics.

[124] Sun W, Luo Z, Lee J, Kim HJ, Lee K, Tebon P, Feng Y, Dokmeci MR, Sengupta S, Khademhosseini A (2019). Adv. Healthc. Mater..

[125] Van Meer BJ, de Vries H, Firth KSA, van Weerd J, Tertoolen LGJ, Karperien HBJ, Jonkheij P, Denning C, Ijzerman AP, Mummery CL (2017). Biochem. Biophys. Res. Commun..

[126] Toepke MW, Beebe DJ (2006). Lab Chip.

[127] Campbell SB, Wu Q, Yazbeck J, Liu C, Okhovatian S, Radisic M (2020). ACS Biomater. Sci. Eng..

[128] Clevers H (2016). Cell.

[129] Fatehullah A, Tan SH, Barker N (2016). Nat. Cell Biol..

[130] Yin X, Mead BE, Safaee H, Langer R, Karp JM, Levy O (2016). Cell Stem Cell.

[131] Kasendra M, Tovaglieri A, Sontheimer-Phelps A, Jalili-Firoozinezhad S, Bein A, Chalkiadaki A, Scholl W, Zhang C, Rickner H, Richmond CA, Li H, Breault DT, Ingber DE (2018). Sci. Rep..

[132] Tao T, Wang Y, Chen W, Li Z, Su W, Guo Y, Deng P, Qin J (2019). Lab Chip.

[133] Kim S, Lee H, Chung M, Jeon NL (2013). Lab Chip.

[134] Haase K, Kamm RD (2017). Regen. Med..

[135] Paek J, Park SE, Lu Q, Park KT, Cho M, Oh JM, Kwon KW, Yi YS, Song JW, Edelstein HI, Ishibashi J, Yang W, Myerson JW, Kiseleva RY, Aprelev P, Hood ED, Stambolian D, Seale P, Muzykantov VR, Huh D (2019). ACS Nano.

[136] Liu C, Oikonomopoulos A, Sayed N, Wu JC (2018). Development.

[137] Cochrane A, Albers HJ, Passier R, Mummery CL, Van Den Berg A, Orlova VV, Van Der Meer AD (2019). Adv. Drug Deliv. Rev..

[138] Esch MB, King TL, Shuler ML (2011). Annu. Rev. Biomed. Eng..

